# Ion Selectivity in the ENaC/DEG Family: A Systematic Review with Supporting Analysis

**DOI:** 10.3390/ijms222010998

**Published:** 2021-10-12

**Authors:** Cédric Vallée, Brendan Howlin, Rebecca Lewis

**Affiliations:** 1 Leverhulme Quantum Biology Doctoral Training Centre, University of Surrey, Guildford GU2 5XH, UK; c.vallee@surrey.ac.uk (C.V.); b.howlin@surrey.ac.uk (B.H.); 2 Department of Chemistry, Faculty of Engineering and Physical Sciences, University of Surrey, Guildford GU2 7XH, UK; 3 School of Veterinary Medicine, Faculty of Health and Medical Sciences, University of Surrey, Guildford GU2 7AL, UK

**Keywords:** ion selectivity, sodium, potassium, lithium, epithelial sodium channel, acid-sensing ion channel, degenerin, FMRF-amide-gated sodium channel

## Abstract

The Epithelial Sodium Channel/Degenerin (ENaC/DEG) family is a superfamily of sodium-selective channels that play diverse and important physiological roles in a wide variety of animal species. Despite their differences, they share a high homology in the pore region in which the ion discrimination takes place. Although ion selectivity has been studied for decades, the mechanisms underlying this selectivity for trimeric channels, and particularly for the ENaC/DEG family, are still poorly understood. This systematic review follows PRISMA guidelines and aims to determine the main components that govern ion selectivity in the ENaC/DEG family. In total, 27 papers from three online databases were included according to specific exclusion and inclusion criteria. It was found that the G/SxS selectivity filter (glycine/serine, non-conserved residue, serine) and other well conserved residues play a crucial role in ion selectivity. Depending on the ion type, residues with different properties are involved in ion permeability. For lithium against sodium, aromatic residues upstream of the selectivity filter seem to be important, whereas for sodium against potassium, negatively charged residues downstream of the selectivity filter seem to be important. This review provides new perspectives for further studies to unravel the mechanisms of ion selectivity.

## 1. Introduction

Ion channels are important pore-forming proteins which control the passive flow of ions (such as sodium (Na^+^), potassium (K^+^), chloride (Cl^−^) and calcium (Ca^2+^)) through the plasma membrane or organelle membrane in living organisms, by shifting from a closed state to an open state. This change in conformation can be mediated by the membrane potential, specific ligands, second messengers, and other stimuli such as mechanical forces, light, etc. Ion channels are widely expressed in different types of cells, and thus play different physiological roles. They can be responsible for the electric signal in the nervous system or they can regulate the cell volume, for example [1]. A disfunction or mutation of some channels may have a significant impact on the health of living beings. Currently, ion channels rank as the second most important target for pharmaceuticals in the market [2,3,4,5]. In order to study these channels, researchers rely on electrophysiological techniques. Thanks to these techniques, they can measure bioelectric parameters such as current flow, conductance, permeability, opening probability, and kinetics (of activation, deactivation, or desensitisation). More recently, with the latest advances in molecular biology, more and more channel structures have been established, and it is possible to elucidate further features of these channels through computational modelling [6,7,8,9,10,11,12].

The Epithelial Sodium Channel/Degenerin (ENaC/DEG) family groups consist of different sodium-selective and amiloride-sensitive channels: the Epithelial Sodium Channel (ENaC); the acid-sensing ion channels (ASICs); the degenerin (DEG) channels; and the FMRF-amide-gated sodium channel (FaNaC). They all share a similar trimeric structure. Despite their similarities, they are expressed in different animals of different phyla, and they display different gating properties. Indeed, the ENaC is constitutively open while the DEG are mechanically activated, and both ASICs and FaNaC are activated by ligands (protons and peptides, respectively) [13,14,15,16]. Although DEG, ASIC, and FaNaC channels are all expressed in the nervous system of their respective host, the ENaC can be found in many different types of cell. ENaC genes were first isolated and identified from rat colon in the 1990s [17,18]. Recently, they have been characterised in the musculoskeletal system, and more precisely in chondrocytes (cells of cartilage) [19].

The Na^+^ and lithium (Li^+^) selectivity over K^+^ is a key characteristic of the functionality of these channels. Alkali metals are known to be difficult to differentiate, but biomolecules have coordinating properties that can be exploited in organic chemistry to improve ion selectivity [20,21,22,23,24]. The ENaC is primarily known to play a role in Na^+^ reabsorption in the kidney, thus it is involved in the regulation of cell volume as well as extracellular fluid volume. Due to its importance, dysfunction of the channel can lead to severe physiological problems. For example, lack of Na^+^ reabsorption in the kidney is associated with the well-known pseudohypoaldosteronism type 1 (reviewed in Kellenberger S. and Schild L., 2002; and Hanokoglu I. and Hanokoglu A., 2016) [14,16]. In cartilage, the ENaC regulates cell volume, and a loss of volume control in chondrocytes is a marker for osteoarthritis [19]. ASICs are known to be involved in the modulation of pain, fear, and memory by mediating influx of excitatory Na^+^ following a decrease in the local extracellular pH [25]. Therefore, several studies have investigated the pore region of these channels, especially in ENaC and ASIC. Even if Li^+^ is not a natural ion, it is known to play significant biological roles and is, for example, used as treatment for bipolar patients [26]. Being more selective to Li^+^, ENaC could possibly be one explanation of some of these roles. Compared to the selectivity of potassium, sodium, and calcium voltage-gated channels, the mechanisms governing ionic selectivity for the ENaC/DEG family are still poorly understood.

Here we systematically review studies focusing on the ion selectivity of the ENaC/DEG family by investigating the pore region of the channels. The aim of this review was to implement reported data regarding ion selectivity with new calculated data based on previous electrophysiological experiments. Together with recent computational data, these results highlight the important determinants of ion selectivity that have been revealed so far and offer new perspectives for further investigation of ion selectivity in these Na^+^-selective trimeric channels [27,28,29,30,31,32,33,34].

## 2. Materials and Methods

### 2.1. Research Question

The research question of this review was: “What are the main components that govern ion selectivity in the ENaC/DEG family?”. The question was developed using the PICO format (Population, Intervention, Comparison, and Outcomes) [35]. Then, the structure of the systematic review followed the guidelines from Preferred Reporting Items for Systematic Reviews and Meta-Analyses (PRISMA) [36].

### 2.2. Search Strategy

The search for literature relevant to our study was conducted in July 2021 on three different databases: PubMed, Scopus, and Google Scholar. The search terms used for PubMed were: “(((ion selectivity[Title/Abstract]) OR (selectivity filter[Title/Abstract])) AND ((epithelial sodium channel[Title/Abstract]) OR (ENaC[Title/Abstract]) OR (Degenerin[Title/Abstract]) OR (ASIC[Title/Abstract]) OR (FaNaC[Title/Abstract])))”. The search terms used for Scopus were “TITLE-ABS-KEY (“ion selectivity” OR “selectivity filter” AND “epithelial sodium channel” OR “ENaC” OR “degenerin” OR “ASIC” OR “FaNaC”)”. Finally, two search terms were used for Google Scholar: “allintitle: selectivity filter ENaC OR degenerin OR DEG OR ASIC OR FaNaC” and “allintitle: ion selectivity ENaC OR degenerin OR DEG OR ASIC OR FaNaC”. A time filter from 1999 to 2021 was applied on each search. Then duplicates were removed.

### 2.3. Study Selection

Results from the search in the literature were further filtered according to rigorously defined exclusion and inclusion criteria. During the screening, only titles and abstracts were analysed to match our criteria. The exclusion criteria were: paper is not an original research paper (i.e., review articles, book chapter, or editorial focus); paper is not in English. The inclusion criteria were: paper is focusing only on a channel from the ENaC/DEG family. Then, if the paper was experimental: paper reports electrophysiology data; paper is focusing on the pore region (TM2 segment). If the paper was computational: paper reports structural data of the pore, paper reports ion selectivity simulations.

### 2.4. Data Collection Process

In this review, three different types of data were collected, each describing different but complementary parameters. First, experimental data from electrophysiological experiments on *Xenopus laevis* oocytes were collected. Within these experimental data, most of them described ion permeability and some of them described residue accessibility. Then computational data were collected.

### 2.5. Additional Analysis

In order to simplify comparison of the data, the sequences from all the channels and subunits that were in the literature were collected. Sequences were found using the Protein database (https://www.ncbi.nlm.nih.gov/protein, accessed on 15 July 2021) of the National Center for Biotechnology Information (NCBI). Here are all the accession numbers for all the sequences found: P37088, P51168, P51170, P51172, NP113736, NP036780, NP058742, NP035454, AAD21245, NP035456, AAY28983, NP033727, NP001029186, AAK20896, AAC47265, P34886, NP001294294, NP505703. Sequences were aligned using either the online tool Clustal Omega Multiple Sequence Alignment (ClustalΩ, https://www.ebi.ac.uk/Tools/msa/clustalo/, accessed on 15 July 2021) or the Align/Superpose tool of MOE 2020.09 software [37].

### 2.6. Calculus and Statistics

The permeability ratio was used as a parameter to describe ion selectivity. For all studies where this was possible, the permeability was calculated using the following Formula (1) [38,39]:

(1) $$\frac{I_{X}}{A}=P_{X}\times E_{test}\times z_{X}\times F^{2}\times \frac{{\left[X\right]}_{out}-{\left[X\right]}_{in}e^{\frac{FE_{test}}{RT}}}{RT}\times \left(1-e^{\frac{FE_{test}}{RT}}\right)$$

With *I_X_* the recorded current (in ampere) for the ion *X*, A the cell surface area, *P_X_* the permeability for the ion *X*, *E_test_* the holding potential (in volt), *z* the valence of the ion, *[X]_out_* and *[X]_in_* the extracellular and intracellular ion concentration, respectively (in molar). *F*, *R*, and *T* have the usual meaning: Faraday constant, gas constant, and temperature, respectively. This formula can be simplified by isolating *P_X_* (2):

(2) $$P_{X}=\frac{I_{X}\times RT}{A\times E_{test}\times z_{x}\times F^{2}\times \left({\left[X\right]}_{out}-{\left[X\right]}_{in}e^{\frac{FE_{test}}{RT}}\right)\times \left(1-e^{\frac{FE_{test}}{RT}}\right)}$$

All reported experiments were carried on the same cells (*Xenopus laevis* oocytes) at approximately the same temperature (room temperature, ~295.65 °K). Currents (*I_X_* and *I_Na_*) used for the formulas were recorded from the same holding potential (*E_test_*). Thus, the permeability ratio can be calculated using the following Formula (3):

(3) $$\frac{P_{X}}{P_{Na}}=\frac{I_{X}\times z_{Na}\times \left({\left[Na\right]}_{out}-{\left[Na\right]}_{in}e^{\frac{FE_{test}}{RT}}\right)}{I_{Na}\times z_{X}\times \left({\left[X\right]}_{out}-{\left[X\right]}_{in}e^{\frac{FE_{test}}{RT}}\right)}\;$$

Reported and calculated permeability ratios were compared by the ANOVA (Analysis Of Variance) test followed by the Tukey post-hoc test HSD (Honestly Significant Difference). The same tests were performed to compare ratios between channels and/or between wild types and variants.

## 3. Results

### 3.1. Search Results

**Figure 1:**
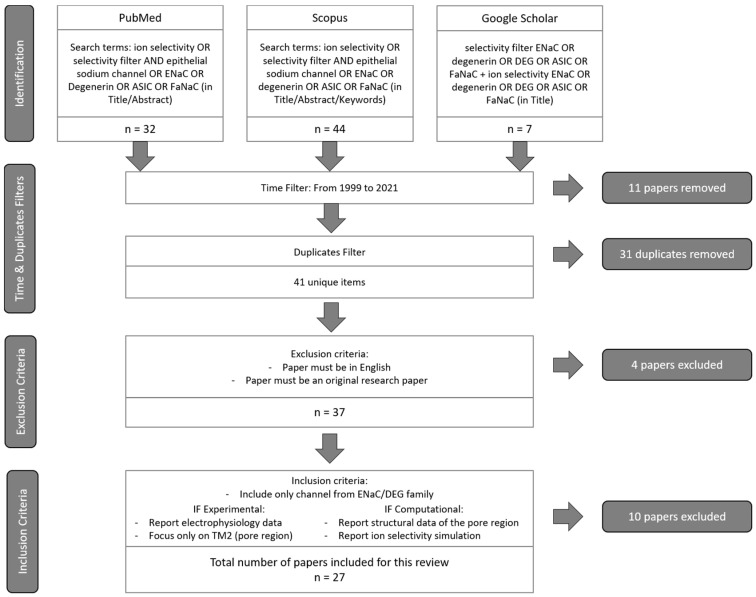
**Flowchart of the search strategy for this review**. Search was carried out in July 2021. The flowchart follows the PRISMA guidelines. Databases are accessible on the following website: PubMed: https://pubmed.ncbi.nlm.nih.gov/, accessed on 15 July 2021; Scopus: https://www.scopus.com/search/form.uri?display=basic#basic, accessed on 15 July 2021; Google Scholar: https://scholar.google.com/, accessed on 15 July 2021.

The flowchart of our search strategy is summarised in Figure 1. In total, there were 83 results for our search terms. First, each result from the different databases was filtered by time (from 1999 to 2021) and duplicates were removed. The remaining 41 articles were then subjected to our exclusion criteria in the first instance, and then to our inclusion criteria in the second instance. In total, 14 other papers were removed in these steps. Finally, 27 relevant papers were obtained for our review (Table 1).

### 3.2. ENaC/DEG Pore Sequences Alignment

**Figure 2:**
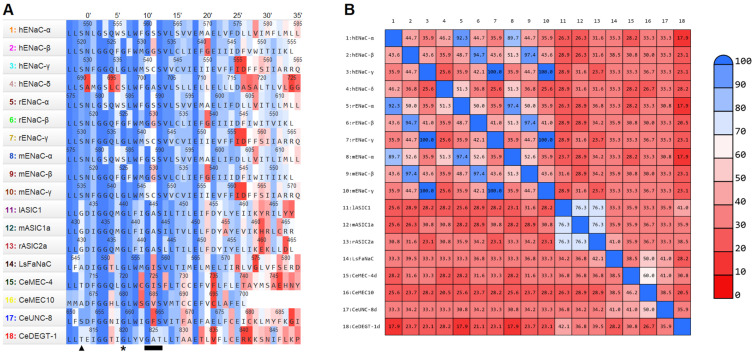
**Sequences of the second transmembrane segment of ENaC/DEG family**. (**A**). Sequence alignment of hENaC-α (P37088), hENaC-β (P51168), hENaC-γ (P51170), hENaC-δ (P51172), rENaC-α (NP113736), rENaC-β (NP036780), rENaC-γ (NP058742), mENaC-α (NP035454), mENaC-β (AAD21245), mENaC-γ (NP035456), lASIC1 (AAY28983), mASIC1a (NP033727), rASIC2a (NP001029186), LsFaNaC (AAK20896), CeMEC-4 (AAC47265), CeMEC-10 (P34886), CeUNC-8 (NP001294294), and CeDEGT-1 (NP505703). Top numbers represent the renumbering systems created by Lynagh T. et al. (2017) [32]. Colours show the percentage of similarity between residues (blue for 100%, red for 0%). The black triangle indicates the degenerin site; the black asterisk indicates the amiloride binding site; and the black rectangle indicates the selectivity filter. (**B**). Identity matrix of the second transmembrane segment of the 18 reviewed channels. Colours show the percentage of identity between sequences (blue for 100%, red for 0%). Both figures were generated using MOE 2020.09 software [37].

There are a total of 12 different channels of the ENaC/DEG family that have been studied among all the articles included in this review. Since the ENaC is heteromeric, these 12 different channels correspond to 18 different subunits. These 18 subunits were first aligned using Clustal Omega online tools. The alignments were then implemented on MOE, and another alignment of the second transmembrane domain of each channel was performed (Figure 2A). Immediately after the alignment, an identity matrix was made on MOE as well (Figure 2B). For simplicity, each residue was renumbered according to the renumbering systems created by Lynagh T. et al. (2017) [32]. The second transmembrane domain was the most conserved segment of the ENaC/DEG. By looking at the alignment, a high degree of similarity between the sequences can be seen, especially the first 20 residues of the sequence. Important residues such as the degenerin site, amiloride binding site, and selectivity filter are part of these first 20 residues. However, by looking at the identity matrix, the degree of identity between sequences is lower. This may reflect the great differences between the phyla of the species which express different types of channels (chordates vs. gastropods vs. nematodes).

### 3.3. ENaC/DEG Permeability Ratios

Ion channels from the ENaC/DEG family are non-voltage-gated ion channels selective for sodium. Within the family, the ENaC distinguishes itself by being almost impermeable to K^+^ and with a higher permeability to Li^+^. For decades, researchers have investigated the ion selectivity of this channel, and members of the same family. Initially, a selectivity filter was proposed for this channel and accepted for the whole family. This selectivity filter consisted of a G/SxS motif, where “x” is a non-conserved residue at position 11′ [40,41,42,45,57]. However, recent studies have been looking at the importance of other residues within the pore regarding this selectivity. Here, all the studies that performed electrophysiological measurements on ENaC/DEG pore single mutation variants using different ions as charge carriers were reviewed.

In order to be able to compare each study, it was necessary to identify a consistent parameter to report. Most of the studies reported the permeability ratio of the tested ion over sodium. Thus, this parameter was used here as well. The permeability is an electrophysiological characteristic that describes the potency of a channel to let a specific species of ions flow through it. Therefore, permeability ratios reflect the ion discrimination of the studied channel. To ensure that all available data are used, the ratios for each study were calculated using Formula (3) [38,39]. Because all experiments were carried on oocytes, for the intracellular concentration in whole cell or cell-attached experiments we used 20 mM, 80 mM, 10^−4^ mM, 5.10^−4^ mM, and 0 mM for Na^+^, K^+^, Ca^2+^, Mg^2+^, and non-natural ions, respectively [58,59]. Other concentrations were reported from the material and methods section of the corresponding paper. When the permeability ratio was provided, both the reported and the calculated ratios were used. In total, 595 permeability ratios of the studied ion over sodium were obtained, with more than 200 for both lithium and potassium (204 and 250, respectively). Among our permeability ratios, 75 calculated ratios were compared to their respective reported ones and all were similar (*p*-value = 0.143). Only comparisons of wild types and single mutation variants are detailed in this review.

#### 3.3.1. Wild-Type Permeability Ratios

Table 2 shows all monovalent ion permeability ratios for the wild-type channels of the ENaC/DEG family. For each channel, lithium and potassium ratios were determined. Although they were analysed, the permeability ratios for divalent and organic ions are not reported here because there were too few data compared to the monovalent ones. As expected, the ENaC αβγ from human, rat, and mouse display the highest Li/Na permeability ratios and the lowest K/Na permeability ratios (reflecting an almost impermeable channel towards this ion). Interestingly, the hENaC αβγ shows a relatively high permeability for caesium and divalent ions compared to potassium. Surprisingly, the hENaC δβγ displays a Li/Na permeability ratio inferior to 1, reflecting a higher selectivity for sodium, and a similar K/Na permeability ratio compared to the hENaC αβγ. This could raise the importance of the α subunit for lithium selectivity in the ENaC αβγ channel. Looking at the sequence alignment of the TM2 of both α and δ subunits, there are two important differences: first, the substitution of almost conserved polar/charged residues to hydrophobic residues at position 0′ (alanine in δ vs. asparagine or aspartate in most channels), and at position 4′ (leucine in δ vs. glutamine in most channels); secondly, a different selectivity filter (“GSS” in α vs. “GAS” in δ) (Figure 2A). The selectivity filter of the hENaC δ subunit is similar to the selectivity filter of the three ASICs, which are themselves more selective to sodium over lithium (except for the mASIC1a + rASIC2a hybrid). As expected again, the monomeric ASICs display Li/Na permeability ratios slightly lower than 1 and K/Na permeability ratios around 0.2. The non-natural hybrid mASIC1a + rASIC2a is slightly more selective for sodium over lithium with Li/Na permeability ratios over 1, with no changes in K/Na permeability ratios. Even if this channel is not representative of any natural channel, mouse and rat ASICs share relatively high identity. As a heteromeric channel, it lacks symmetry within the pore which is also a characteristic of the heterotrimeric ENaC, which is the most selective channel for lithium.

The DEG channels from *C. elegans* (CeMEC-4d, CeMEC-4d + CeMEC-10, CeUNC-8d, and CeDEGT-1d) show different properties. The first two possess Li/Na and K/Na permeability ratios relatively similar to those of ASICs previously described. The CeUNC-8d shows a higher Li/Na permeability ratio but still lower than those of ENaC αβγ. It is also the only channel to possess a K/Na permeability ratio between 0.5 and 1. The most surprising result from Table 2 is the permeability ratios of CeDEGT-1d which is more permeable to potassium and caesium than sodium and lithium. By comparing the sequences from the alignment, the most striking difference is the “GAT” motif for the selectivity filter. It is the only channel of the alignment to not possess the conserved serine residue (position 12′) of the G/SxS motif (Figure 2A). Although the residue is different, both serine and threonine share a similar functional group: hydroxyl. That could point out a larger importance of the structure rather than the function. It is also important to mention that CeDEGT-1d also lack the conserved pre-TM1 HG motif (not shown in our sequence alignment).

The HaFaNaC displays unique permeability ratios, with a slightly higher permeability for lithium over sodium and a permeability for potassium slightly lower than the potassium permeability in ASICs.

#### 3.3.2. Permeability Ratios of ENaC/DEG Variants

Permeability ratios of lithium and potassium over sodium for different functional variants of rENaC αβγ, mENaC αβγ, lASIC, mASIC1a, rASIC2a, and CeMEC-4d channels were reported. Among all the substitutions investigated in every study reviewed, we focused on residues where at least one significant change in either lithium or potassium permeability ratio was reported in any of the previously cited channels. Surprisingly, this analysis identified significant changes for 25 residues (3′, 5′, 6′, 7′, 8, 9′, 10′, 11′, 12′, 14′, 15′, 16′, 17′, 18′, 19′, 20′, 21′, 23′, 24′, 25′, 26′, 27′, 30′, and 33′). Because some reported ratios were not statistically analysed in their respective studies, values were determined for permeability ratios of variants which are most likely significantly different from the wild type. To simplify the determination of these values, wild-type permeability ratios were statistically compared. Because most of the variants were generated using the rat and mouse ENaC and ASIC, wild-type ratios from these channels were used as references. It appears that there were not significant differences between rENaC αβγ and mENaC αβγ (*p*-value = 0.65, *n* = 16), nor between mASIC1a and rASIC2a (*p*-value = 0.43, *n* = 14). Comparisons of the statistically significant ratios to percentages of their respective wild-type ratios were performed, and there were no differences between significant reported results and 30% of wild type (*p*-value: 0.721). Thus, our ratio was characterised in two different ways. First, important decreases/increases of the permeability ratios for changes from wild type by ± 30% were determined (less than 1.23 or more than 2.29 for ENaC lithium ratios; more than 0.01 for ENaC potassium ratios; less than 0.66 or more than 1.23 for ASIC lithium ratios; less than 0.17 or more than 0.32 for ASIC potassium ratios; less than 0.56 or more than 1.04 for MEC-4d lithium ratios; less than 0.14 or more than 0.26 for MEC-4d potassium ratios). Because both rENaC and mENaC are impermeable to potassium, the value was determined arbitrarily based on significant results from papers. Secondly, permeability ratios which describe a change in ion selectivity were determined (less than 1 for ENaC lithium ratios; more than 1 for ENaC potassium ratios; more than 1 for both ASIC lithium and potassium ratios; more than 1 for both MEC-4d lithium and potassium ratios). It is important to note that over the 25 residues reported as important for ion permeability, 14 of them were also reported as important for functionality of the channel, with some substitution leading to a non-functional or very low-conductance channel. This characteristic highlights the difficulties of studying ion selectivity in this family as most of the important residues for selectivity seem also to be involved in functionality of the channel.

##### ENaC

Table 3 shows the permeability ratios for different variants of the ENaC pore. For the wild type, the channel is twice as permeable to lithium than sodium and is almost not permeable to potassium. The pore region is one of the most conserved regions in the ENaC/DEG family, and mutations along this region could result in channels with low conductance or even non-functionality. Nevertheless, some mutations are supported and make it possible to exhibit the electrophysiological relevance of different residues. Changes in permeability support the idea that the studied residue could play a role in ion permeability. If these changes are very large and the ratio is then reversed, it could even demonstrate a role in ion selectivity. Based on the physical and chemical properties of both the substituted and variant residues, many hints on the channel conduction are drawn from these parameters.

Here variants that significantly change permeability ratios for ENaC are first reported. Table 3 shows that most changes of permeability ratios impact either lithium or potassium ratios. Very few variants change permeability ratios for both ions. Among this minority, some even change the selectivity of the channel and will be discussed later in this section. The two variants that significantly change both lithium and potassium permeability ratio without changing selectivity are D25′C (αENaC) and γD26′C. Interestingly, both residues are aspartate, a negatively charged residue. For D25′ (αENaC), when the residue is substituted to glutamate, another negatively charged residue, or substituted to asparagine, its neutral counterpart, the permeability does not change. Moreover, when D25′ (αENaC) is substituted to lysine, a positively charged residue, only the potassium ratio changes, but it changes more than with the substitution to cysteine. Surprisingly, no changes of permeability ratios were observed for D25′C (βENaC) variants. These results support the idea of an asymmetric pore, as D26′ (γENaC) residue is most likely to play a similar role than D25′ (αENaC), and also suggest the importance of the negative charge for potassium selectivity.

Regarding lithium permeability ratios, lots of variants change ratios by either increasing or decreasing. Interestingly, a pattern can be extracted for Table 3. Indeed, it appears that most variants located before and within the G/SxS motif selectivity filter result in a decrease of lithium permeability ratios, and even in a change of selectivity. On the other hand, most variants located after the selectivity filter result in an increase on lithium permeability. Those observations could support the idea of two different barriers for ions located within ENaC pores.

Regarding potassium permeability ratios, as the wild type is almost not permeable to potassium, the analysis is mainly focused on how permeable to this ion the ENaC becomes with the variants. The main changes occur for the selectivity filter variants, with some that even change the selectivity. Other changes occur after the selectivity filter, and especially at the well conserved E18′ residue and at both D25′ (αENaC) and D26′ (γENaC) residues, as mentioned earlier. Interestingly, those residues are all negatively charged residues.

Variants that reverse the permeability ratio over sodium, and thus describe a change in ion selectivity, are reported here. Not surprisingly, selectivity is affected when residues of the G/SxS selectivity filter are substituted. Unfortunately, this region is quite sensitive to mutations, and most variants are not functional, so no consensus can be extracted from analysis. However, it is important to note that the only residue that changes potassium and sodium selectivity is the well conserved S12′ residue. Interestingly, depending on the variants, either the lithium selectivity or the potassium selectivity change, but not both for the same variant.

Out of the selectivity filter, only two variants were reported to change lithium selectivity. These variants are W5′C and W8′C (αENaC). Interestingly, both residues at location 5′ and 8′ are tryptophan, an aromatic residue. Aromatic residues have the particularity to possess aromatic rings in which lots of π-electrons can be found. Cations are known to interact with π-electrons, resulting in π-cation interactions. These results might reflect the importance of π-cation interactions for lithium selectivity.

##### ASIC

Table 4 shows the permeability ratios for different variants of the ASIC pore. Contrarily to the ENaC, wild-type ASICs are more selective to sodium than both lithium and potassium. Although more selective for sodium, lithium permeability ratios are almost 1 for both mASIC1a and rASIC2a, describing a weak discrimination between these two ions species and thus a relatively similar selectivity. Similar to the ENaC, the pore region of ASICs is quite sensitive to mutations, and maybe even more sensitive than ENaC. Since they can be functional as a monomer, one mutation in the DNA sequence will result in variants with three different amino acids from the wild-type channels. That is reflected by the difficulty of obtaining functional variants, especially when the G/SxS selectivity filter is targeted for substitutions. Following the same procedure as for the ENaC, we analysed the changes in permeability ratios for ASIC.

In contrast to the ENaC, for ASIC it seems that most of the variants are affecting the potassium permeability rather than the lithium permeability ratios. As was observed for the ENaC, only few variants change both lithium and potassium permeability ratios, and similar to ENaC again, most of the changes are so important that the selectivity itself changes as well. Only the A11′C variant, which is a residue of the selectivity filter, alters both permeability without interfering with the selectivity.

For the lithium permeability ratio, because the value is close to 1, any slight increase will lead to a change in selectivity. Thus, only significant decreases of lithium permeability ratios will be discussed here. Only two of the reported variants exhibited a decrease in lithium permeability ratios: G10′Cx1 (lASIC1) and A11′C (mASIC1a). Interestingly, both variants are located within the selectivity filter. These results support the importance of the selectivity filter regarding permeabilities for lithium and sodium.

For potassium permeability ratios, it is difficult to establish a consensus as variants of similar or adjacent residues can have opposing impacts on the permeability ratio, with one variant increasing it and the next one decreasing it, or vice versa. However, one variant specifically caught our attention: the A11′α (mASIC1a). This variant is unusual but is of particular interest as it changes the chemical properties of the backbone peptide bond between residue 10′ and 11′ without altering the sidechain of the residue at position 11′. By changing the peptide bond from amide to ester, the A11′α variant modifies the properties of the upstream residue (G10’) by lowering the electrostatic potential of the oxygen of its carbonyl group. The result observed from this variant is a decrease of potassium permeability. This observation suggests that electrostatic properties of the atom from residues in the pore that are interacting with the ions could be a key element for understanding ions permeation and probably selectivity as well.

In ASICs, lithium and sodium permeabilities are quite similar. Even though the channels are more selective to sodium, a small increase of permeability to lithium or a small decrease of permeability to sodium will lead to a change in selectivity. Consequently, many variants were reported with reversion of lithium selectivity against sodium. Among all of them, only a few have lithium permeability ratios significantly different from the wild type: residue 6′, residue 7′, and residue 18′. All those three residues are well conserved in the ENaC/DEG family. These same residues are also reported with a reversion of potassium permeability ratios, and thus impact potassium selectivity as well. The variants responsible for these changes are almost identical for both ion species. This could suggest a similar mechanism of ion discrimination for this channel.

##### DEG Channels

For the DEG channels, among the 25 interesting residues of the pore selected for this review, only three variants were investigated: residues 7′, 11′, and 14′. These residues were previously reported as important for both ion permeation and selectivity in ASIC. The same was reported for residue 11′ for the ENaC, but residue 7′ seems unlikely to be important for permeability and residue 14′ has not been investigated. For the DEG, the L7′C does not change permeability ratios for lithium nor for potassium, like the ENaC. The I11′C variant does not change the permeability ratio for lithium but does change it for potassium. This result differs slightly from the results observed for both ENaC and ASIC. Finally, the residue 14′ seems to be important for ion permeability, as both lithium and potassium permeability ratios change for the L14′C variant. This result supports the observation made for L14′ variants in ASIC, with different impacts depending on the mutation.

### 3.4. ENaC/DEG Pore Lining Residues Accessibility

In order to further characterise the pore of ENaC/DEG family channels, many studies have investigated the accessibility of single residues within the second transmembrane segment by systematically substituting the residue by cysteine and using either MTS reagents or divalent ions like zinc (Zn^2+^) or cadmium (Cd^2+^). Here the accessibility of residues previously reported as important for ion selectivity to MTS reagents or divalent ions are reviewed. Among all seventeen residues reported to be important for ion selectivity, only nine were studied for accessibility in both ENaC and ASIC (residues 5′, 6′, 7′, 8′, 9′, 10′, 11′, 12′, and 14′).

#### 3.4.1. Pre-Amiloride Binding Site Region

The only residue prior to the amiloride binding site that has been shown to change ion selectivity when mutated is the residue 5′. This residue is not really conserved in the ENaC/DEG family. The substitution of this residue to cysteine in the α-subunit of mENaC (W5′C) results in a channel that is almost twice as permeable to sodium than lithium. Contrarily, substitution of this residue to cysteine in mASIC1a (M5′C) does not change the lithium permeability ratio but does change the potassium permeability ratio without disturbing the selectivity of the channel.

In mENaC, the W5′C variant is affected by both MTSEA and cadmium at millimolar concentration. External treatment of this variant with the reagent and the ion induced a significant increase of amiloride-sensitive current. However, external treatment of this variant to MTSET does not change the amiloride-sensitive current amplitude. These interactions suggest that the sidechain of this residue is accessible to small reagents or ions but is unlikely to be oriented straight towards the centre of the pore. Unfortunately, in ASIC, although permeability ratios have been determined in mASIC1a, the M5′C variant in lASIC1 provided a non-functional channel. The accessibility of this residue could not be analysed for this channel.

#### 3.4.2. The Amiloride Binding Site

The residue at position 6′ is well conserved in the ENaC/DEG family and is known to be the amiloride binding site. Permeability ratios of variants of this residue have not been determined in the ENaC as substitution of this residue will drastically change the amiloride sensitivity, which is crucial for electrophysiologic characterisation of this channel. In mASIC1a, which is activated by low extracellular pH, the substitution of this residue to cysteine (G6′C) results in a channel more selective to lithium with an increase of the potassium permeability ratio.

In mENaC, the 6′ residue of the α-subunit is less important for the amiloride binding than the 6′ residue of both β- and γ-subunits. Thus, the impact of reagents and cadmium on the substitution of the non-conserved serine to cysteine (S6′C) were investigated. External application of millimolar concentration of MTSEA and MTSET to this variant results in an almost total inhibition of amiloride-sensitive current. These results suggest that the hydroxyl side chain of the serine residue is lined up directly toward the pore and supports the fact that interaction of an external compound with this residue would inhibit ENaC. Surprisingly, in mASIC1a the G6′C variant is only modified by millimolar concentration of MTSEA but is not modified by millimolar concentration of MTSET, suggesting a slightly different pore size between ENaC and ASIC at this region.

Moreover, the same S6′C substitution in the α-subunit of ENaC is inhibited by both divalent ions Zn^2+^ and Cd^2+^ at sub-millimolar concentrations. Based on the voltage dependencies of these blockages, the residue 6′ as been localised between 3% to 12% of the electric field in a region extracellular to the selectivity filter. Unfortunately, like for the residue 5′, the G6′C variant in lASIC1 results in a non-functional channel.

#### 3.4.3. Region between the Amiloride Binding Site and the Selectivity Filter

The region between the amiloride binding site and the selectivity filter constitutes three residues. They have all shown a change in ion selectivity for some mutations. This region is not really conserved, only the residue 7′ and residue 8′ are quite conserved with leucine and aromatic residues, respectively. The impact of variants in this region varies from ENaC to ASIC.

In mENaC, no changes in amiloride-sensitive current were observed when the L7′C variant was treated by external MTS reagents. However, inhibition of currents was observed when the same variant was treated by external cadmium at millimolar concentration. In lASIC, the L7′C variants were functional and were characterised. Firstly, L7′C variants were treated by external MTSEA either in closed state or in open state (low pH). Interestingly, channel activity of this variant is modified when MTSEA is applied externally in an open state, but no modification was observed when applied externally in a closed state. Conversely, for internal MTSEA treatments modifications were observed when applied in a closed state, but not when applied in an open state. These results suggest that the 7′ residue is important for, or located near a region important for, gating in ASIC.

Even if mutations of residue 8′ have been reported as important for ion permeability for both ENaC and ASIC, especially for lithium permeability in ENaC, the W8′C and F8′C variants seem to not be affected by MTS reagent. These results suggest that the sidechain of this residue is unlikely to be accessible to the chemical compound. Finally, cysteine variants at position 9′ do not seem to be available for MTS reagents.

#### 3.4.4. The Selectivity Filter Region

The selectivity filter of ENaC/DEG family was determined as the G/SxS motif from residue 10′ to 12′. Not surprisingly, variants at these positions change ion selectivity. These residues are crucial for ENaC/DEG activity, and it is difficult to study by mutagenesis as many variants will be non-functional or will have very small conductance. In fact, the accessibility of cysteine variants could only be investigated on the less conserved residue at position 11′, or by using concatemeric constructs with mutation in only one of the three monomers of ASIC.

Application of MTSEA on a single monomer variant G10′C in lASIC1 does not seem to change activity of the channel. This result could reflect a region too narrow for the MTS reagent or simply show that the sidechain of this residue is not available and consequently the carbonyl group is likely to be oriented towards the pore. For the S11′C variant in the α-subunit of ENaC, amiloride currents seem to be inhibited when treated by MTSEA but not when treated by either MTSET or cadmium. Knowing that MTSET is a bigger MTS reagent than MTSEA, these results give supplementary evidence about the size of the pore at this location. Surprisingly, cadmium seems to not bind with the cysteine of the S11′C variant, although smaller than MTSEA. This observation may involve a selectivity based on the charge (MTSEA has only one positive charge, cadmium has two). Unfortunately, in lASIC1 A11′C variants were not functional enough to be characterised for accessibility and no MTS reagent investigation could be driven for S12′C variants in ENaC or in ASIC.

The only study for S12′C variants was done on the α-subunit of ENaC with cadmium. Sub-millimolar inhibition was observed in S12′C variant and surprisingly in S12′D variant as well. Aspartate and glutamate are acidic residues and thus are negatively charged. Even though cadmium is known to have a high affinity for the thiol group of cysteines, carboxyl group of aspartates can also bind divalent ions with high affinity (like calcium, for example). However, both S12′N and S12′A are inhibited by millimolar and centimollar concentrations of cadmium, respectively. Although the asparagine residue is polar, alanine is hydrophobic and is unlikely to bind cysteine. These results suggest that the inhibition of S12′C variant by cadmium could be due to another residue rather than the S12′C substitution itself. This observation supports the one made for 11′ variants regarding selectivity based on both sizes and charges at this location.

#### 3.4.5. Post-Selectivity Filter Region

Among all other residues shown to change ion selectivity in the post-selectivity filter region, only the residue 14′ has been investigated for accessibility. This residue is in the ENaC/DEG family, being a non-aromatic hydrophobic residue for each sequence and especially a leucine for most of the time. This residue was the last residue which changes ion selectivity to be reported for accessibility. In the α-subunit of ENaC, like for the S11′C variant, the L14′C variant is only inhibited when treated by MTSEA but is not when treated by either MTSET or cadmium, supporting the idea of size and charge exclusion. However, in lASIC1, L14′C seems to not be inhibited by MTSEA treatment either by external or internal application of the MTS reagent in both open and closed states. This may reveal slight conformation differences between ENaC and ASIC.

### 3.5. Computational Studies on ENaC/DEG Pore Region

#### 3.5.1. Channel Structure

**Figure 3:**
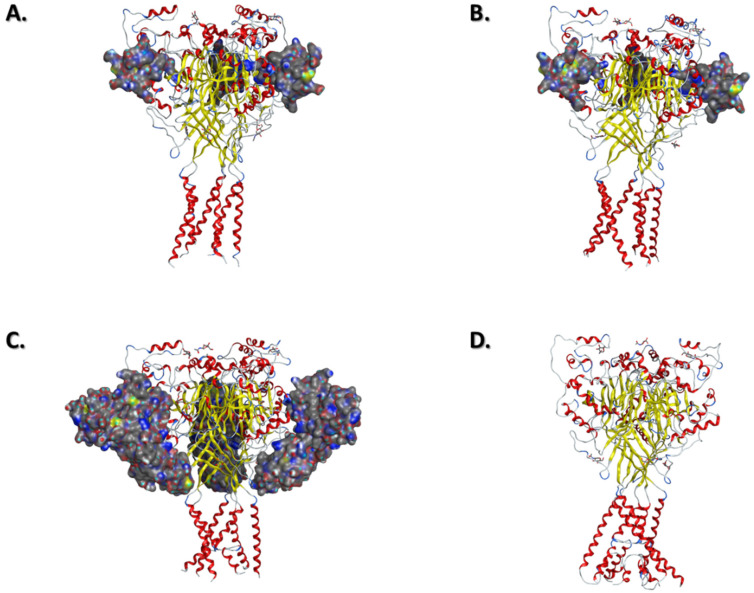
**Available ASIC structures with different configuration of the pore**. (**A**). Structure of cASIC1a in complex with PcTx1 at low pH (PDB: 4ZF0). (**B**). Structure of cASIC1a in complex with PcTx1 at high pH (PDB: 4ZF1). (**C**). Structure of cASIC1a in complex with MitTx (PDB: 4NTW). (**D**). Structure of cASIC1a with HG loop solubilized by SMA at low pH (PDB: 6VTK).

Baconguis I. and Guouax E. (2012) reported two different structures of the cASIC1a in high-pH and low-pH concentrations in complex with a spider toxin: Psalmotoxin (PcTx1) (Figure 3A,B) [28]. This toxin acts as an agonist on cASIC1a by activating the current and decaying the steady state of the channel, even without acidic stimulation. Interestingly, the architectures of the pore differ in both structures and so does ion selectivity. In the high-pH PcTx1 complex, the pore is symmetrical and larger with a smallest pore diameter of about 10 Å (at position D0′) and cannot discriminate Li^+^, Na^+^, K^+^, Cs^+^, or NMDG. In the low-pH PcTx1 complex, the pore is asymmetrical and smaller with a constriction point of about 5 to 7 Å (at position L7′) and is selective for both sodium and lithium over potassium. These data coincide with the experimental results described above.

Another structure of an open configuration of the cASIC1a stabilised by a potent snake toxin agonist (MitTx) has been reported by Baconguis I. et al. (2014) (Figure 3C) [29]. The cASIC1a-MitTx complex is selective for lithium and sodium over potassium, like the natural channel once activated by acidic stimuli. Although more symmetrical, this new structure shares some similarities with the low-pH Na^+^ selective cASIC1a-PcTx1 complex but provides further details on the GAS selectivity filter. Indeed, in this new open structure the GAS motif consists of an extended narrow region of the channel pore with a diameter of about 7 Å at position G10′. Moreover, electrostatic mapping of the cASIC1a-MitTx complex shows that the potential charges within the pore are mainly negative due to the presence of negatively charged residues and carboxyl groups from conserved glycines (G3′, G6′ and G10′) lining the pore. Together with the previous study, these structural results strengthen results from experiments regarding the importance of conserved residues in the pore for ion selectivity.

More recently, Yoder N. and Gouaux E. (2020) presented a new structure of cASIC1a solubilised in styrene maleic acid (SMA) (Figure 3D) [34]. This new structure revealed the existence of a re-entrant loop in the pre-TM1 region. This re-entrant loop has the purpose of presenting the HG motif, preserved in most if not all ENaC/DEG channels, that was lacking in all preceding structures. Interestingly, the HG motif of this structure is located directly beneath the selectivity filter and forms a second constriction point that can be considered as a second barrier for ion conduction.

#### 3.5.2. Computational Simulations

Thanks to the different structures of ASIC, people started to look at the ion selectivity through computation models. They mostly investigated the differences between sodium and potassium by looking at the Free Energy Calculations on different models. The free energy of a system shows how spontaneous the reaction is, allowing numerical comparisons to be made between different reactions.

In a first approach, Dudev T. and Lim C. modelled small trimer selectivity filter-like models and looked at the difference based on whether the ion is coordinated to the carbonyl oxygen from an amide group of a residue or with the oxygen from a hydroxyl group of a residue (like the conserved serine of the selectivity filter) [27,30,31]. They increased the dielectric constant to make the system more similar to a biological system. They found negative free energies when sodium was replacing potassium within filter models in most of the cases, representing a sodium selectivity. These free energies were stronger for the filter model with oxygen from amide group coordinating the ion and were highly dependent on the hydration number of the substituting ion. They also tested selectivity for sodium against calcium and the free energies were even higher. Altogether, these results suggest that the ion selectivity of ENaC/DEG family is most likely to be explained by coordination of sodium through carbonyl oxygens, and unlikely to be explained by oxygens from hydroxyl groups.

In another approach, Lynagh T. et al. (2017) used the previously described open structure of the cASIC1a channel (PDB: 4NTW) for molecular dynamics simulation [32]. The protein was embedded in a bilayer lipid membrane and solvated with 150 mM of NaCl or KCl. The free energy profiles for both ions were generated by umbrella sampling. Surprisingly, they did not find any particular differences in the free energies at the GAS motif coordinates between simulations with sodium or with potassium. However, they did find differences in other positions, notably L7′ and E18′. These results were further supported with homology models of rASIC2a using the same open cASIC1a structure (PDB: 4NTW) as a template [33]. The same results were observed, especially at position E18′, which seems to be the main residue responsible for sodium vs. potassium selectivity in ASIC.

## 4. Discussion

Ion selectivity in ion channels has been investigated for decades. The mechanisms underlying such an effective property have been relatively well described for voltage-gated ion channels, and especially for the bacterial potassium channel KcsA [60,61,62,63,64,65,66,67,68,69]. However, for non-voltage-gated ion channels, such as the channels of the ENaC/DEG family, the mechanisms underlying ion selectivity are still poorly understood and a matter of debate. Here, the literature focusing on ion selectivity for the ENaC/DEG family was systematically reviewed and the principal components that are most likely to govern this mechanism were highlighted.

Ion selectivity is such an important property of ion channels that mutations in the pore region and/or the selectivity filter often result in loss of function channels, especially within the ENaC/DEG family. This characteristic reveals how difficult it is to investigate ion selectivity in channel mutants via conventional methods, such as electrophysiology. Thus, comparing wild-type sequences of the pore region from channels of the same family is a good preliminary approach. In the ENaC/DEG family, the second transmembrane domain of the channel, also known as the pore region, is the most conserved region. However, even if it is the most conserved region, the percentage between subunits of different channels is still low (less than 50% in most cases) although the percentage of similarity is high. Knowing that all the ENaC/DEG channels are selective to sodium but share different electrophysiological properties, and thus different degrees of selectivity, suggests that both residue function and structure are important, with function hypothetically determining sodium selectivity and structure hypothetically determining the degree of selectivity. To validate these hypothesises, permeability ratios between wild types and functional mutants were compared and the accessibility studies of relevant residues for selectivity were collected.

### 4.1. Lithium vs. Sodium Selectivity

The ENaC/DEG family is known to display a high permeability to lithium, one of the smallest alkali metals. For each channel analysed in this review, the Li/Na permeability ratio was over 0.5, describing relatively similar permeabilities. Some channels (the ENaC αβγ, the mASIC1a + rASIC2a hybrid, the CeUNC-8d and the HaFaNaC) are even lithium selective, with a Li/Na permeability ratio between 1 and 2.

Through these mutational studies, important residues for lithium selectivity have been identified for mouse and rat ENaC αβγ and ASICs. For the ENaC, residues 5′, 8′, 10′, 11′, and 12′ and for the ASIC, residues 6′, 7′, and 18′ have been reported to be important for lithium selectivity. Not surprisingly, residues of the selectivity filter (10′, 11′, and 12′) are involved for the ENaC. Although these key residues are different, this could be explained by the fact that study of the ENaC cannot be carried out with a mutation on the residue 6′ as it is the amiloride binding site, and being a constitutively open channel, affinity to a selective blocker like amiloride and benzamil is imperative for studying this channel. Moreover, as ASICs are homotrimeric channels, a mutation in one subunit results in three mutations on the channel, which is not supported when the mutations are localised in the selectivity filter. Thus, one can argue that both the amiloride binding site and selectivity filter are the most likely key components for both functionality and ion selectivity for both channels. Another interesting feature of the lithium selectivity is the importance of tryptophan residues in the ENaC (W5′ and W8′). Both residues are aromatic in the α subunit, and a change in selectivity was observed when they were substituted to cysteine. Even if there is no change in lithium selectivity for the other subunits of ENaC or with other substitution of amino acids, there is still a significant decrease in Li/Na permeability ratios. Interestingly, wild-type ASICs do not have aromatic residues at position 5′ and unfortunately residue 8′F was not highly studied, however, it appears that substituting 8′F to tryptophan (like in ENaC, 8′W) does not perturb lithium selectivity. In addition, ASICs do not have aromatic residues at position 9′ either, but by substituting isoleucine to phenylalanine (I9′F), an aromatic residue, we can observe a change of lithium selectivity, although the shift of the permeability ratio does not seem significant. These results highlight a potential involvement of aromatic residues in the selectivity for lithium. Even if accessibility experiments on cysteine substitutions do not support the idea that these residues are lined up the centre of the pore, a potential link with the presence of numerous π electrons located on aromatic residues cannot be excluded. Indeed, it is known that cations can interact with these electrons, and these interactions are called π-cation interactions [70]. It is also known that the strength of these interactions is different based on both the type of aromatic ring and the type of cation interacting [71,72].

### 4.2. Sodium vs. Potassium Selectivity

Sodium selectivity over potassium is central for ENaC/DEG channel function, which is characterised by K/Na permeability ratios below 0.5. This ratio is even almost null for the ENaC, describing a virtually impermeable channel for potassium. So far, the key element governing this selectivity was thought to only be the residues of the selectivity. However, the mechanisms behind it and the involvement of other residues are still a matter of debate.

In a similar way as for lithium, this review highlights the role of pore residues by using reported mutational and accessibility studies. As expected, substitutions of residues in the selectivity filter are responsible for the reversal of potassium selectivity in ENaC, but surprisingly in ASICs the K/Na permeability ratio changes significantly without reversing. Once again, mutations in the selectivity filter of homomeric ASICs are more difficult to study as it affects all three subunits of the channel and researchers resort to concatemeric constructs. However, in ASICs, substitution of other residues results in a reversal of K/Na ratio: residues G6′, L7′, L14′ and E18′. As observed for lithium, the amiloride binding site seems to be key for both function and ion selectivity. Interestingly, a huge increase in K/Na permeability ratio can be observed upon substitution of a negatively charged residue (E18′ and D21′) for a neutral or positively charged in the ENaC. This might reveal an important role of negatively charged residues for sodium selectivity over potassium. Indeed, recent computational studies on ASIC showed a more discriminating site at the negatively charged residue compared to the selectivity filter [32,33]. Moreover, voltage-gated sodium channels also display negatively charged residues in their selectivity filter (DEKA), which are essential for sodium selectivity over potassium as well [73,74,75,76,77,78,79]. Unfortunately, conserved residues L7′ and L14′ have been under-investigated in ENaC for ion selectivity. By looking at accessibility experiments, substitution of both L7′ and L14′ by cysteine in the ENaC results in inhibited channels when treated by cadmium and MTSEA, respectively. In ASIC, only the L7′C mutant is inhibited by MTSEA. This again highlights differences between ENaC and ASIC, however knowing that the side chain of leucine is hydrophobic, if it is lining the pore, it is unlikely to interact directly with the ions, thus these conserved residues might be of a structural importance, especially with their proximity to the crucial region of the pore: the amiloride binding site and selectivity filter.

### 4.3. Perspectives

This review combined experimental and computational studies and identified potential key elements for ion selectivity in the ENaC/DEG family. Due to the difficulties in studying these key elements experimentally and the development of physical and chemical analysis techniques, computational tools are proving to be useful resources for the study of ionic selectivity. More and more protein structures have been determined, as is the case for the ENaC [80,81]. With the support of the data reported in this review and the emerging new data, the mystery surrounding the selectivity in ion channels tends to become clearer. 

As mentioned previously, aromatic residues seem important for lithium selectivity, whereas they seem to not play any role regarding potassium selectivity. To inspect these parameters, aromatic residues can be substituted with other aromatic molecules with different π-electron clouds and engineered channels can be studied by electrophysiology. Otherwise, π-cation interactions could be investigated computationally using Quantum Mechanics/Molecular Mechanics (QM/MM) simulations [82]. Moreover, it has been shown that the structure of the channel pore plays a central role in ion selectivity in the ENaC/DEG family. While it is known that structure is a fundamental feature of all proteins, the contribution of the latter is still not fully understood regarding ion selectivity in ion channels.

Finally, channels of the ENaC/DEG family share a surprising similarity in structure with purinoreceptor P2X channels: trimeric channels with two transmembrane segments and a large extracellular domain [83]. P2X channels have been excluded from this review as they do not belong to the ENaC/DEG family, nevertheless exploring proteins with similar structure and similar function can lead to new perspectives.

## 5. Conclusions

In this review with supporting analysis, we confirmed the importance of the G/SxS selectivity filter and other highly conserved residues (amiloride binding site and HG motif) for both the ion selectivity and function of channels of the ENaC/DEG family. Furthermore, we showed that the mechanisms for Li^+^ vs. Na^+^ selectivity and Na^+^ vs. K^+^ selectivity are most likely different and involve aromatic and negatively charged residues, respectively. Overall, this review highlights the importance of ion discrimination in ion channels but also highlights the physicochemical complexity of the mechanisms underlying this selectivity in the ENaC/DEG family.

## Figures and Tables

**Table 1:** **Table summarising the papers analysed for this review**. All 27 papers are listed here by date of publication.

Publication	Channel Studied	Experimental vs. Computational	Main Outcome of the Study
Kellenberger S. et al., 1999 [40]	ENaC (αβγ)	Experimental	Na^+^ selectivity vs. Li^+^, K^+^, Rb^+^, Cs^+^, Ca^2+^, Mg^2+^, Sr^2+^ and Ba^2+^ in single point mutation variants.
Kellenberger S. et al., 1999 [41]	ENaC (αβγ)	Experimental	Na^+^ selectivity vs. Li^+^ and K^+^ in single point mutation variants.
Sheng S. et al., 2000 [42]	ENaC (αβγ)	Experimental	Na^+^ selectivity vs. Li^+^ and K^+^ in channel variants.
Sheng S. et al., 2001 [43]	ENaC (αβγ)	Experimental	Pore accessibility in cysteine channel variants with MTS reagent and Cd^2+^.
Ji HL. et al., 2001 [38]	ENaC (αβγ)	Experimental	Na^+^ selectivity vs. Li^+^, K^+^ and NMDG^+^ in channel variants.
Sheng S. et al., 2001 [44]	ENaC (αβγ)	Experimental	Na^+^ selectivity vs. Li^+^ and K^+^ in single point mutation variants.
Kellenberger S. et al., 2001 [45]	ENaC (αβγ)	Experimental	Na^+^ selectivity vs. Li^+^, K^+^, Rb^+^, Cs^+^ and organic cations in single point mutation variants.
Ji HL. et al., 2004 [39]	ENaC (αβγ) & ENaC (δβγ)	Experimental	Na^+^ selectivity vs. Li^+^ and K^+^ in α- and δ-subunit channel variants.
Carattino M.D. et al., 2005 [46]	ENaC (αβγ)	Experimental	Channel gating in TM2 single point mutation variants.
Sheng S. et al., 2005 [47]	ENaC (αβγ)	Experimental	Pore accessibility in cysteine channel variants by Cd^2+^ inhibition.
Ji HL. et al., 2006 [48]	ENaC (αβγ) & ENaC (δβγ)	Experimental	Na^+^ selectivity vs. Li^+^, K^+^, Cs^+^, Ca^2+^ and Mg^2+^ in α- and δ-subunit expressing cell.
Takeda AN, et al., 2007 [49]	ENaC (αβγ)	Experimental	Pore accessibility in single point mutation variants by Cd^2+^ inhibition.
Dudev T. & Lim C., 2010 [27]	ENaC selectivity filter models	Computational	Na^+^ selectivity vs. K^+^ by ion exchange free energy calculations.
Li T. et al., 2011 [50]	ASIC1	Experimental	Na^+^ selectivity vs. Li^+^, K^+^ and Cs^+^ in single point mutation variants.
Li T. et al., 2011 [51]	ASIC1	Experimental	Na^+^ selectivity vs. Li^+^, K^+^ and Cs^+^ in single point mutation variants.
Carattino M.D. & Della Vecchia M.C., 2012 [52]	ASIC1a	Experimental	Na^+^ selectivity vs. Li^+^, K^+^, Rb^+^, Cs^+^ and pore accessibility with MTS reagent in single point mutation variants.
Baconguis I. & Guouax E., 2012 [28]	ASIC1a	Experimental & Computational	Structure of ASIC1a and spider toxin complex.
Baconguis I. et al., 2014 [29]	ASIC1a	Experimental & Computational	Structure of ASIC1a and snake toxin complex.
Dudev T. & Lim C., 2014 [30]	ENaC selectivity filter models	Computational	Na^+^ selectivity vs. K^+^ and Ca^2+^ by ion exchange free energy calculations.
Dudev T. & Lim C., 2015 [31]	ASIC1a selectivity filter models	Computational	Na^+^ selectivity vs. K^+^ and Ca^2+^ by ion exchange free energy calculations.
Lynagh T. et al., 2017 [32]	ASIC1a	Experimental & Computational	Na^+^ selectivity vs. Li^+^ and K^+^ in single point mutation variants & Na^+^ and K^+^ free energy profiles
Yang XN. et al., 2017 [53]	FaNaC	Experimental	Pharmacology and Na^+^ selectivity vs. Li^+^, K^+^ and Cs^+^.
Shi S. et al., 2018 [54]	DEG (MEC-4 & MEC-10)	Experimental	Gating properties and Na^+^ selectivity vs. Li^+^ and K^+^ in channel variants.
Yang L. & Palmer L.G., 2018 [55]	ENaC (αβγ)	Experimental	Na^+^ selectivity vs. Li^+^ and K^+^ voltage dependence inhibition in channel variants.
Lynagh T. et al., 2020 [33]	ASIC1a & ASIC2a	Experimental & Computational	Na^+^ selectivity vs. Li^+^ and K^+^ in single point mutation variants & Na^+^ and K^+^ free energy profiles
Fechner S. et al., 2020 [56]	DEG (DEGT-1, UNC-8, MEC-4 and MEC10)	Experimental	Na^+^ selectivity vs. Li^+^, K^+^, Cs^+^ and NMG^+^ in wild-type channels
Yoder N. & Gouaux E., 2020 [34]	ASIC1	Experimental & Computational	New structure of the channel with HG re-entrant loop

**Table 2:** **Permeability ratios of tested ion over Na^+^ for wt ENaC/DEG channels**. Averages of ratios obtained from papers and by calculation. The number of ratios used is shown in brackets.

Channel	*P_Li_*/*P_Na_*	*P_K_*/*P_Na_*	*P_Rb_*/*P_Na_*	*P_Cs_*/*P_Na_*
hENaC αβγ	1.640 (6)	0.018 (4)	n.d.	0.255 (2)
rENaC αβγ	1.695 (6)	N.P. (6)	N.P. (1)	N.P. (1)
mENaC αβγ	1.823 (2)	N.P. (2)	n.d.	n.d.
hENaC δβγ	0.616 (4)	0.051 (2)	n.d.	n.d.
lASIC1	0.529 (2)	0.208 (2)	n.d.	0.009 (2)
mASIC1a	0.901 (4)	0.276 (4)	0.060 (1)	0.036 (4)
rASIC2a	0.994 (2)	0.224 (2)	n.d.	N.P. (2)
mASIC1a + rASIC2a	1.100 (2)	0.243 (2)	n.d.	N.P. (2)
CeMEC-4d ^a^	0.859 (2)	0.230 (2)	n.d.	0.062 (2)
CeMEC-4d ^a^ + CeMEC-10	0.736 (1)	0.170 (1)	n.d.	n.d.
CeUNC-8d ^a^	1.523 (2)	0.609 (2)	n.d.	0.263 (2)
CeDEGT-1d ^a^	0.638 (2)	1.725 (2)	n.d.	1.553 (2)
HaFaNaC ^b^	1.200 (4)	0.178 (4)	n.d.	N.P. (1)

n.d.—not determined. N.P.—not permeable to referred ion. ^a^ DEG channels with the corresponding degenerin (“d”) mutation. ^b^ HaFaNaC shares about 65% identity with LsFaNaC. HaFaNaC’s sequence is available on Yang X-N., et al. (2007) [53].

**Table 3:** **Permeability ratios of tested ion over Na^+^ for ENaC single mutations variants**. Ratios that are different from wild type by ±30% are represented in italics (considerate as “significant”); and ratios that describe change in ion selectivity are represented in bold.

**Position**	**Channel**	**Mutant**	***P_Li_*** **/*P_Na_***	***P_K_*** **/*P_Na_***	**Position**	**Channel**	**Mutant**	***P_Li_*** **/*P_Na_***	***P_K_*** **/*P_Na_***
	rENaC αβγ	wt	1.695	N.P.	**12**′	rENaC-α	S589C	1.286	*0.227*
	mENaC αβγ	wt	1.823	N.P.			S589D	1.415	*0.313*
**3′**	mENaC-α	S580C	2.174	*0.060*			S589G	1.231	*0.040*
**5′**	mENaC-α	W582C	***0.549***	N.P.			S589N	***0.665***	*0.710*
**7′**	mENaC-α	L584C	1.840	N.P.			S589Q	***0.563***	*0.244*
**8′**	rENaC-α	W585A	1.296	N.P.			S589H	*1.188*	***1.031***
		W585C	***0.997***	N.P.		rENaC-β	S531A	1.405	*0.010*
		W585E	*1.196*	N.P.		rENaC-γ	S543A	2.193	N.P.
		W585R	*1.096*	N.P.		mENaC-α	S589A	***0.877***	*0.567*
	rENaC-β	W527C	*1.096*	N.P.			S589C	1.634	***1.781***
		W527E	1.495	N.P.			S589T	***0.857***	N.P.
	rENaC-γ	W539A	*1.096*	N.P.	**15**′	mENaC-α	S592C	*2.997*	N.P.
		W539E	1.296	N.P.	**16**′	mENaC-α	V593C	*2.677*	N.P.
**9′**	mENaC-α	F586C	*1.089*	N.P.	**17**′	mENaC-α	V594C	*3.087*	0.002
**10′**	rENaC-α	G587A	***0.997***	*0.010*	**18**′	mENaC-α	E595C	1.602	*0.111*
		G587S	1.595	*0.010*	**19**′	mENaC-α	M596C	*3.048*	N.P.
	rENaC-β	G529A	***0.299***	N.P.	**20**′	mENaC-α	A597C	*3.067*	N.P.
		G529S	2.193	*0.222*	**21**′	mENaC-α	E598C	*3.048*	N.P.
		G529C	***0.698***	*0.061*	**23**′	mENaC-α	I600C	*2.774*	N.P.
		G529D	2.153	*0.192*	**24**′	mENaC-α	F601C	*2.794*	0.006
		G529R	*2.392*	*0.020*	**25**′	mENaC-α	D602C	*2.364*	*0.115*
	rENaC-γ	S541A	***0.199***	N.P.			D602K	1.862	*0.419*
		S541G	*3.090*	N.P.			D602N	2.057	N.P.
	mENaC-α	G587A	***0.966***	*0.810*			D602E	2.129	N.P.
		G587C	1.713	*0.233*		mENaC-β	D544C	1.867	N.P.
**11′**	rENaC-α	S588A	1.395	*0.010*	**26**′	mENaC-α	L603C	*2.423*	N.P.
		S588I	***0.698***	N.P.		mENaC-γ	D562C	*2.386*	*0.011*
	rENaC-β	G530A	*1.196*	*0.010*	**27**′	mENaC-α	L604C	*2.344*	N.P.
	rENaC-γ	C542A	1.894	N.P.	**30**′	mENaC-α	T607C	*2.598*	N.P.
**12′**	rENaC-α	S589A	***0.947***	*0.056*	**33**′	mENaC-α	M610C	2.247	*0.020*

N.P.—not permeable to potassium.

**Table 4:** **Permeability ratios of tested ion over Na^+^ for ASIC single mutations variants**. Ratios that are different from wild type by ±30% are represented in italic (considered as “significant”) and ratios that describe change in ion selectivity are represented in bold.

Position	Channel	Mutant	*P_Li_*/*P_Na_*	*P_K_*/*P_Na_*	Position	Channel	Mutant	*P_Li_*/*P_Na_*	*P_K_*/*P_Na_*
	lASIC1	wt	0.529	0.208	**14′**	mASIC1a	L446C	**1.050**	***1.057***
	mASIC1a	wt	0.901	0.276			L446A	0.957	*0.989*
	rASIC2a	wt	0.994	0.224			L446I	**1.205**	0.180
**5**′	mASIC1a	M437C	0.750	*0.493*		rASIC2a	L445A	0.880	*0.400*
**6**′	mASIC1a	G438C	***1.400***	*0.960*			L445I	0.889	*0.341*
		G438A	**1.180**	***1.290***	**15′**	mASIC1a	T447S	**1.128**	0.206
**7**′	mASIC1a	L439A	***1.285***	***1.255***	**16′**	mASIC1a	V448T	**1.104**	0.225
		L439Ax1 ^a^	**1.106**	*0.131*			V448A	0.916	0.193
		L439Ax2 ^b^	**1.205**	*0.136*	**17′**	mASIC1a	L449A	**1.153**	0.215
		L439V	**1.056**	0.218			L449I	0.947	*0.160*
		L439I	0.858	*0.122*	**18′**	mASIC1a	E450D	0.906	*0.611*
	rASIC2a	L438A	0.887	0.273			E450Q	***1.382***	***1.549***
**8**′	mASIC1a	F440L	0.957	*0.164*			E450Qx1 ^a^	0.738	0.220
		F440W	0.886	0.235			E450Qx2 ^b^	0.950	*0.431*
**9**′	mASIC1a	I441A	0.916	0.189		rASIC2a	E449Q	0.949	*1.130*
		I441F	**1.011**	*0.167*	**19′**	mASIC1a	L451A	**1.153**	0.187
**10**′	lASIC1	G443Cx1 ^a^	*0.342*	0.264			L451I	**1.022**	0.233
**11**′	mASIC1a	A443C	*0.600*	*0.593*	**20′**	mASIC1a	F452L	**1.080**	0.175
		A443G	0.958	*0.062*	**21′**	mASIC1a	D453E	**1.116**	*0.377*
		A443S	**1.113**	0.200			D453N	**1.104**	*0.645*
		A443α ^c^	0.958	*0.062*	**25′**	mASIC1a	E457D	0.978	0.202
**12**′	mASIC1a	S444Ax1	0.683	*0.143*			E457Q	**1.080**	0.240
		S444Ax2	0.828	*0.136*					

^a^ Concatemeric channel with mutation in only one subunit. ^b^ Concatemeric channel with mutation in two subunits. ^c^ Lactate variant (with ester substitution at the amide function of the peptide bond between residue 11′ and 12′).
